# Using the theory of planned behaviour to understand the adoption of vegetarianism among females in Saudi Arabia

**DOI:** 10.3389/fpubh.2025.1566712

**Published:** 2025-05-14

**Authors:** Areej Ali Alkhaldy

**Affiliations:** Department of Clinical Nutrition, Faculty of Applied Medical Sciences, King Abdulaziz University, Jeddah, Saudi Arabia

**Keywords:** vegetarianism, vegetarian diet, plant-based diets, vegetarian, vegan, theory of planned behavior

## Abstract

**Background:**

The adoption of vegetarian diets is increasing worldwide, including among females in Saudi Arabia. This increase has not yet been explained. The present study explores the factors influencing the intention to follow a vegetarian diet among Saudi women using the theory of planned behavior (TPB) framework.

**Materials and methods:**

An online self-administered questionnaire was distributed between July 2023 and January 2024. A total of 998 Saudi females participated, including both vegetarians (*n* = 417, 41.8%) and non-vegetarians (*n* = 581, 58.2%), who answered questions relating to their sociodemographic characteristics, awareness and perceived understanding of vegetarian diets, awareness of and adherence to dietary guidelines, dietary habits, and TPB predictors. Multivariable linear regression was used with dietary guideline awareness, body mass index, self-rated perceived weight, socioeconomic status index, and the TPB constructs as predictors to explain the intention to follow a vegetarian diet (outcome).

**Results:**

Attitude (standardized *β*-coefficient = 0.29, *p* < 0.001), subjective norms (standardized *β*-coefficient = 0.10, *p* < 0.001), and perceived behavioral control (standardized β-coefficient = 0.08, *p* = 0.03) were significantly and positively associated with the intention to follow a vegetarian diet. However, dietary guideline awareness did not significantly influence the intention (standardized *β*-coefficient = −0.04, *p* = 0.1). Socioeconomic status index was negatively correlated with the intention to adopt a vegetarian diet (standardized *β*-coefficient = −0.073, *p* = 0.018). Body mass index showed no significant correlation with vegetarian diet intention (standardized *β*-coefficient = −0.05, *p* = 0.091).

**Conclusion:**

TPB constructs (attitude, subjective norms, and perceived behavioral control) and socioeconomic status influence the intention to adopt a vegetarian diet, although awareness of dietary guidelines and body mass index do not.

## 1 Introduction

Vegetarianism is becoming increasingly popular among various cultures and ethnic groups worldwide, including in Saudi Arabia (1, 2). In general, vegetarianism refers to refraining from consuming one or more categories of animal-based foods. Vegetarian diets can be further categorized into vegan (entirely plant based), lacto-vegetarian (consuming dairy products but not eggs, meat, or fish), lacto-ovo-vegetarian (consuming dairy products and eggs but not meat or fish), pesco-vegetarian (consuming fish and seafood but not other types of meat, with or without dairy products and eggs), and pollo-vegetarian (consuming poultry but not other types of meat or fish) (3). Individuals may adopt vegetarian diets for a variety of reasons, including ethical considerations, potential health benefits, personal preferences, environmental concerns, or religious or cultural beliefs (4–6). The potential health benefits and risks vary among the different vegetarian diets. However, despite the fact that following strict and unbalanced vegetarian diets may lead to some health complications, both the Academy of Nutrition and Dietetics (formerly the American Dietetic Association) and Dietitians of Canada hold the position that vegetarian diets are nutritionally appropriate, are suitable for individuals in various stages of life, and could offer health benefits for the prevention and treatment of diseases (7).

Accompanying the growing popularity of vegetarian diets, the amount of research into such diets has been increasing since 2015 as part of the drive to realize the 17 Sustainable Development Goals defined by the United Nations in its 2030 Agenda for Sustainable Development (8). This is relevant in terms of food production and the eating habits of consumers because at least nine of these goals are related to global food systems becoming more sustainable (8). However, research into vegetarian diets has so far been mixed; some studies have focused on the health benefits and environmental sustainability of vegetarian diets (9–12), whereas others highlighted concerns about inadequate nutrition and potential risks (13).

A recent meta-analysis of 76 prospective cohort studies including 2,230,443 participants reported that adherence to a plant-based diet was inversely associated with the risks of type 2 diabetes, cardiovascular disease, cancer, and all-cause mortality (14). However, other studies have reported multiple complications, and severe deficiencies may be exacerbated if supplements are not utilized or nutritional recommendations and guidelines for vegetarians are not followed. For example, it has been reported that deficiencies of certain nutrients, including protein, calcium, iron, zinc, vitamin D, and vitamin B_12_, could lead to lower bone mineral density, fatigue, tingling, poor cognition, and poor digestion (15).

At the global level, previous studies have reported the prevalence rates of vegetarianism to range from 1 to 40% across countries. The rate of vegetarianism is estimated to be 1% in China, Italy, and New Zealand (16–18); about 2.7% in France (19); 4.3% in Germany (20); 5% in the US (21); 3.2% in the UK (22); 8% in Canada (23); 33% in South Asia (24); and 40% in India (25). However, there is a lack of data of prevalence rates of vegetarianism in Middle Eastern and Gulf countries, including Saudi Arabia (26), and therefore little is known about the number of followers of vegetarian diets and their nutritional behavior in these countries, including Saudi Arabia. A few studies have investigated the knowledge and attitudes about vegetarianism and associated factors among the Saudi population, examining the proportions of people following different vegetarian diets and identifying their motives for doing so (2, 27, 28). These studies found that health issues, ethical concerns, and environmental concerns were the most common motivators for individuals adopting vegetarian diets, with the majority of Saudis following omnivore diets. The majority of participants in these studies were female (<80%). In addition, there are few studies conducted in countries with similar socio-cultural or religious characteristics, such as the United Arab Emirates (29), Kuwait (30), Jordan (31), and Lebanon (32). However, these studies aim to explore a range of topics, including the alignment of sustainable food choices, assessment of knowledge and attitudes toward food sustainability, comparison of health impacts between vegetarians and non-vegetarians, and an examination of the reasons for consuming a vegetarian diet in relation to health impacts, although no attempt was made to understand and predict the behaviour in relation to following a vegetarian diet using a theoretical approach.

The theory of planned behavior (TPB) is a widely accepted framework for understanding human behaviors and can be applied to study dietary behavior and design interventions for encouraging behavioral changes (33–35). Another popular approach is the transtheoretical model of behavior change (TMBC), which is used to identify people’s stage of change and tailor interventions to changes in their behavior (36). In the present study, we employed the TPB model rather than the TMBC because our aim was to explore the factors influencing whether people follow vegetarian diets. In addition, the TPB model has been adopted in numerous previous studies that aimed to understand people’s intention to follow certain diets (37–39). The TPB model identifies intention as a central concept and explores explicit factors explaining the intention to perform the behavior in question. In the TPB model, intention is regarded as the most powerful psychological determinant of whether someone follows a vegetarian diet, which is predicted by three other psychological constructs: attitude towards vegetarian diets (i.e., whether the person is in favor of following a vegetarian diet), subjective norms (i.e., the social pressure from others to follow or not follow a vegetarian diet), and perceived behavioral control over the behavior (i.e., assessing the individual’s self-efficacy and their beliefs about the controllability of following a vegetarian diet) (33, 34).

In Saudi Arabia, a meat-based diet is standard (40). There is a lack of data to explain the increasing popularity of vegetarian diets and their particular popularity among females (>80% of participants) (28, 41). Therefore, the purpose of the present study was to explore the factors governing the intention of females in Saudi Arabia to follow a vegetarian diet using the TPB framework. This study aims to address the following research question: What factors influence the intention of Saudi females to adopt a vegetarian diet within the framework of the TPB? Understanding the behavior of Saudi females with respect to following a vegetarian diet is important because females have nutritional requirements related to menstruation, pregnancy, and breastfeeding. The findings of this study could assist with the design and implementation of strategies to educate women about the importance of following a nutritionally appropriate diet, including supplementation where necessary, and improve the engagement of healthcare professionals in raising awareness and reducing potential complications arising from unbalanced vegetarian diets.

## 2 Materials and methods

### 2.1 Study design and participants

This cross-sectional study was conducted between July 2023 and January 2024. among Saudi females aged 18 years or older. The inclusion criteria were as follows: (1) aged at least 18 years, (2) females, (3) participants who self-identified as vegetarian and those who self-identified as non-vegetarian, and (4) resident in Saudi Arabia. The exclusion criteria were as follows: (1) age < 18 years, (2) males, and (3) Saudis resident outside Saudi Arabia. Participants were asked to complete an online questionnaire prepared in Google Forms, ensuring that all questions were required to be answered, which resulting in no missing data. The questionnaire was distributed using the snowball sampling method via social media platforms namely WhatsApp and X. At the beginning of the online questionnaire, participants were informed about the aims of the study, inclusion criteria, estimated time to complete the questionnaire, and confidentiality. Participants were required to provide consent for their participation. The study was approved by the Biomedical Ethics Research Committee of King Abdulaziz University (No. 522–20).

### 2.2 Sample size

An online sample size calculator (Epi Info) (42) was utilized to estimate the required sample size using data obtained from the Saudi General Authority for Statistics (43) based on an estimated total of 12,496,629 Saudi females (≥18 years old). The effective sample size was calculated to be *n* = 385, with a 99% confidence interval and a hypothesized 50% frequency of outcome factor in the population.

### 2.3 Questionnaire instrument

The data for this study were self-reported using an online self-administered questionnaire. The questionnaire was constructed following an extensive literature review (38, 44–46). The initial questionnaire was developed in English and then translated into Arabic using the Brislin backtranslation method (47, 48). The questionnaire was assessed for face and content validity by eight experts in clinical nutrition and public health nutrition, and several of the questions and answers were modified based on the obtained feedback. Prior to participant recruitment, the final version of the questionnaire was tested among six people outside of the fields of nutrition and medicine to assess its readability and clarity. The final questionnaire consisted of five sections with a total of 53 questions and required 20 min to complete.

#### 2.3.1 Sociodemographic and background characteristics

This section included 18 questions about age, marital status, educational level, work status, income, field of study, medical diagnoses, smoking habits, physical activity level, food allergies, supplement use, and sources of dietary information. The self-reported height (centimeters) and weight (kilograms) were also collected from participants to allow calculation of their body mass index (BMI). In addition, there were two questions about the participants’ perception of their weight and diet quality. The final question in this section asked participants whether they have relatives or friends who are vegetarians.

#### 2.3.2 Awareness and perceived understanding of vegetarian diets

This section included two questions aimed at assessing the participants’ awareness and perceived understanding of vegetarian diets: ‘Have you heard of vegetarian diets?’ (possible answers: ‘yes’ or ‘no’), and ‘Do you understand what the term “vegetarian diets” means?’ (possible answers: ‘no to moderate understanding’ or ‘good to excellent understanding’).

#### 2.3.3 Awareness of and adherence to dietary guidelines

Participants were asked whether they were aware of four well-established dietary guidelines, namely, Healthy Vegetarian Patterns (USA), the Healthy Food Palm (Saudi Arabia), MyPyramid (USA), and My Plate (Saudi Arabia), and whether they followed these guidelines (possible answers: ‘yes’ or ‘no’).

#### 2.3.4 Dietary assessment

This section assessed the dietary habits of participants using a short food frequency list. The list included common foods, namely, fruit, vegetables, nuts, seeds, legumes, fruit juice, white bread, wholemeal/mixed-grain bread, breakfast cereals, cooked cereals, red meat, white meat, fish/seafood, eggs, and dairy products. For each food item, participants were asked to report their frequency of consumption in the last 6 months by choosing one of the following options: ‘daily’, ‘5–6 days per week’, ‘1–4 days per week’, ‘less than once per week’, or ‘never’.

### 2.4 The theory of planned behavior

The TPB model was used to evaluate vegetarian behavior and the intention to follow a vegetarian diet. The adopted TPB model included the intention to follow a vegetarian diet, attitude towards following a vegetarian diet, subjective norms, and perceived behavioral control over vegetarian behavior. The Cronbach’s alpha test of internal consistency showed that the 3-item behavioral control questions, 4-item subjective norms questions, and 3-item attitude questions had good internal consistency (Cronbach’s alpha = 0.892, 0.871, and 0.922, respectively).

Attitude assessed the participants’ beliefs about the expected outcomes of following a vegetarian diet. The answers were ranked on a seven-point scale to determine whether each participant had a favorable or unfavorable attitude towards the behavior. Participants were asked how ‘bad’ to ‘good’, ‘harmful’ to ‘beneficial’, and ‘unpleasant’ to ‘pleasant’ it was to follow a vegetarian diet. The responses to these items were scored between one and seven, with higher scores reflecting a positive attitude to following a vegetarian diet.

Subjective norms were also assessed using a seven-point scale. Participants were asked to rate how much they felt each of four groups of people (friends, family, health experts, and colleagues) wanted them to follow a vegetarian diet, from ‘not at all’ (score: 1) to ‘to a very great extent’ (score: 7). Higher scores reflected a greater social pressure to follow a vegetarian diet.

Perceived behavioral control was also evaluated using a seven-point scale for the following three items: ‘How much personal control do you feel you have eaten a vegetarian diet in the next year?’ (possible answers: ‘very little control’ to ‘extreme/complete control’), ‘To what extent do you see yourself as capable of following a vegetarian diet in the next year?’ (possible answers: ‘not very capable’ to ‘very capable’), and ‘How easy or difficult do you think it would be to follow a vegetarian diet in the future?’ (possible answers: ‘very difficult’ to ‘very easy’). The responses to these questions were scored between one and seven, with higher scores indicating a greater level of control over the target behavior.

Finally, intention was assessed by asking the participants to record how much they agreed or disagreed with the following statement: ‘I intend to follow a vegetarian diet in the next year’ (possible answers: ‘strongly disagree’ to ‘strongly agree’). The answers were again scored on a seven-point scale, with higher scores indicating a stronger intention.

### 2.5 Statistical analysis

Data were analysed using SPSS Statistics version 27.0 (SPSS Inc., Chicago, IL, United States), and path analysis was performed with the IBM SPSS AMOS structural equation modeling program (version 22). Descriptive statistics including numbers and percentages were calculated for qualitative variables (participant characteristics, awareness, and perceived understanding of vegetarian terms and dietary guidelines), while the medians and interquartile ranges (IQRs) were calculated for quantitative variables. Group differences between vegetarians and non-vegetarians were assessed using chi-square tests for categorical data to evaluate the distribution of sociodemographic characteristics. In addition, Pearson’s chi-square tests were performed to examine independence of two nominal variables: awareness levels and perceived understanding, as well as the awareness of dietary guidelines and adherence levels among vegetarians and non-vegetarians. Non-parametric statistics (Mann–Whitney tests) were used to compare differences in consumption frequencies for various food items and to evaluate the constructs of the TPB among vegetarians and non-vegetarians. For structural equation modeling, the normality assumptions and the equality of variances assumptions for all the metric scores were analyzed using histograms, Kolmogorov–Smirnov statistical normality tests, Mardia’s statistics and the residuals analyses under the structural equation modeling environment. Additionally, the collinearity and multicollinearity diagnostics were also carried out (VIF and Tolerance indices).

Path analysis was conducted to assess the hypothesized TPB model, using multiple linear regression analyses to determine the psychological constructs of the TPB (attitude score, subjective norms score, perceived behavioral control score, dietary guideline awareness score, BMI, self-rated perceived weight, and socioeconomic status index) that influence the intention of participants (dependent variable) towards following a vegetarian diet. The frequency of consumption (fruit, vegetables, nuts, seeds, legumes, fruit juice, white bread, wholemeal/mixed-grain bread, breakfast cereals, and cooked cereals) was used as a dependent variable in a linear regression analysis to assess participants’ attitudes, subjective norms, and perceived behavioral control to understand its impact on the intention to follow a vegetarian diet. The dietary guideline awareness score was based on five questions: one question (the second question) detailed in the materials and methods subsection 2.3.2 and four questions described in subsection 2.3.3 of the materials and methods, while the socioeconomic status index included the income, educational level, marital status, and working status. Pearson correlation tests were used to assess the correlations between the measured metric variables. Multivariable binary logistic regression analysis was applied to identify the statistically significant predictors for the odds of participants being vegetarians or non-vegetarians. The associations between the predictor (independent) variables and the analysed outcomes in the binary logistic regression analysis were expressed as multivariable-adjusted odds ratios (ORs) with their associated 95% confidence intervals. The structural equation modeling path model analysis method was used to evaluate the statistical significance of the predictors for the frequency of plant-based food intake score. The path model fit was assessed with the chi-square standardized method, the comparative fit index (CFI), Normed fit Index (NFI), and the Tucker–Lewis index (TLI). For the global goodness of fit, the root mean square error of approximation (RMSEA) was used; studies have shown that RMSEA values of <0.08 can be regarded as indicating good fit (49). The path model standardized regression coefficients and path model standardized covariances (allowed ones) are quoted in this manuscript. The alpha significance level was evaluated at an alpha level of 0.050.

## 3 Results

### 3.1 Sociodemographic and background characteristics of the participants

A total of 998 female participants completed the online questionnaire. Table 1 summarizes their sociodemographic and background characteristics. More than half of the participants reported being non-vegetarian (58.2%), whereas the others reported being vegetarian (41.8%). Most of the participants were aged between 18 and 24 years (60.3%), and this age group accounted for approximately four-fifths of the participants who considered themselves vegetarian (79.4%) and 46.6% of those who considered themselves non-vegetarian (*p* < 0.001). Most of the participants were single (70.6%), and this group accounted for the majority of the participants who considered themselves vegetarian (90.6%) and 56.3% of those who considered themselves non-vegetarian (*p* < 0.001).

**Table 1:** Sociodemographic characteristics of the participants (*n* = 998).

	Overall (*n* = 998)	Vegetarian *n* (%)^a^ 417 (41.8)	Non-vegetarian *n* (%)^a^ 581 (58.2)	*p* value^b^
Age, years				
18–24	602 (60.3)	331 (79.4)	271 (46.6)	* < 0.001
25–39	276 (27.7)	82 (19.7)	194 (33.4)	
40–59	112 (11.2)	4 (1.0)	108 (18.6)	
≥60	8 (0.8)	0 (0.0)	8 (1.4)	
Marital status				
Single	705 (70.6)	378 (90.6)	327 (56.3)	* < 0.001
Married	258 (25.9)	28 (6.7)	230 (39.6)	
Divorced	25 (2.5)	8 (1.9)	17 (2.9)	
Widowed	10 (1.0)	3 (0.7)	7 (1.2)	
Educational level				
Less than high school	11 (1.1)	5 (1.2)	6 (1.0)	* < 0.001
High school	245 (24.5)	129 (30.9)	116 (20.0)	
University	655 (65.6)	266 (63.8)	389 (67.0)	
Higher degree	87 (8.7)	17 (4.1)	70 (12.0)	
Work status				
Student	548 (54.9)	297 (71.2)	251 (43.2)	* < 0.001
Employed	240 (24.0)	69 (16.5)	171 (29.4)	
Retired	21 (2.1)	0 (0.0)	21 (3.6)	
Unemployed	189 (18.9)	51 (12.2)	138 (23.8)	
Study/work outside Saudi Arabia				
Yes	170 (17.0)	49 (11.8)	121 (20.8)	* < 0.001
No	828 (83.0)	368 (88.2)	460 (79.2)	
Income, Saudi riyals per month (U.S. dollars per month)				
No income	291 (29.2)	138 (33.1)	153 (26.3)	* < 0.001
<2000 (<540 USD)	345 (34.6)	176 (42.2)	169 (29.1)	
2000–4,000 (540–1,066 USD)	107 (10.7)	48 (11.5)	59 (10.2)	
4,001–7,000 (1067–1866 USD)	67 (6.7)	19 (4.6)	48 (8.3)	
7,001–10,000 (1867–2,665 USD)	75 (7.5)	18 (4.3)	57 (9.8)	
10,001–15,000 (2666–4,000 USD)	65 (6.5)	11 (2.6)	54 (9.3)	
>15,000 (>4,000 USD)	48 (4.8)	7 (1.7)	41 (7.1)	
Field of study				
Literature	316 (31.7)	131 (31.4)	185 (31.8)	*0.004
Scientific	433 (43.4)	190 (45.6)	243 (41.8)	
Medical	33 (3.3)	14 (3.4)	19 (3.3)	
Nutrition	54 (5.4)	9 (2.2)	45 (7.7)	
Other healthcare professions	100 (10.0)	49 (11.8)	51 (8.8)	
No specific field	62 (6.2)	24 (5.8)	38 (6.5)	
Body mass index^c^				
Underweight	112 (11.2)	69 (16.5)	43 (7.4)	* < 0.001
Normal	560 (56.1)	279 (66.9)	281 (48.4)	
Overweight	197 (19.7)	40 (9.6)	157 (27.0)	
Obese	129 (12.9)	29 (7.0)	100 (17.2)	
Medical diagnoses				
Yes	371 (37.2)	135 (32.4)	236 (40.6)	*0.008
No	627 (62.8)	282 (67.6)	345 (59.4)	
Smoking habits				
Ex-smoker	48 (4.8)	21 (5.0)	27 (4.6)	0.94
Yes	70 (7.0)	30 (7.2)	40 (6.9)	
No	880 (88.2)	366 (87.8)	514 (88.5)	
Physical activity				
Fairly inactive	507 (50.8)	160 (38.4)	347 (59.7)	* < 0.001
Moderately active	345 (34.6)	167 (40.0)	178 (30.6)	
Very active	146 (14.6)	90 (21.6)	56 (9.6)	
Food allergies				
Yes	185 (18.5)	93 (22.3)	92 (15.8)	*0.010
No	813 (81.5)	324 (77.7)	489 (84.2)	
Self-rated perceived weight				
Underweight	109 (10.9)	49 (11.8)	60 (10.3)	* < 0.001
Normal	472 (47.3)	253 (60.7)	219 (37.7)	
Overweight	339 (34.0)	86 (20.6)	253 (43.5)	
Obese	78 (7.8)	29 (7.0)	49 (8.4)	
Diet quality				
Poor	201 (20.1)	47 (11.3)	154 (26.5)	* < 0.001
Fair	307 (30.8)	103 (24.7)	204 (35.1)	
Good	256 (25.7)	106 (25.4)	150 (25.8)	
Very good	157 (15.7)	109 (26.1)	48 (8.3)	
Excellent	77 (7.7)	52 (12.5)	25 (4.3)	
Supplement use				
Yes	457 (45.8)	223 (53.5)	234 (40.3)	* < 0.001
No	541 (54.2)	194 (46.5)	347 (59.7)	
Supplements used if yes				
Vitamin D	177 (17.7)	79 (18.9)	98 (16.9)	–
Vitamin B_12_	158 (15.8)	139 (33.3)	19 (3.3)	
Multivitamins and minerals	100 (10.0)	34 (8.2)	66 (11.4)	
Iron	85 (8.5)	22 (5.3)	63 (10.8)	
Omega-3	44 (4.4)	16 (3.8)	28 (4.8)	
Vitamin C	22 (2.2)	08 (1.9)	14 (2.4)	
Calcium	19 (1.9)	04 (1.0)	15 (2.6)	
Sources of dietary information				
Family members	219 (21.9)	36 (8.6)	183 (31.5)	* < 0.001
Friends/peers/colleagues	161 (16.1)	37 (8.9)	124 (21.3)	
Books/magazines	148 (14.8)	101 (24.2)	47 (8.1)	
Internet websites	617 (61.8)	322 (77.2)	295 (50.8)	
Media	69 (6.9)	25 (6.0)	44 (7.6)	
Social media	457 (45.8)	259 (62.1)	198 (34.1)	
Healthcare professionals^d^	350 (35.1)	128 (30.7)	222 (38.2)	
Organizations^e^	214 (21.4)	103 (24.7)	111 (19.1)	
Relatives/friends are vegetarian				
No	436 (43.7)	186 (44.6)	250 (43.0)	* < 0.001
Yes	367 (36.8)	190 (45.6)	183 (31.5)	
Unsure	195 (19.5)	41 (9.8)	154 (26.5)	

a Data are presented as numbers and percentages.

b *p* values were obtained by chi-square tests.

c Calculated based on self-reported weight and height.

d Doctors, nurses, dietitians, etc.

e Ministry of Health, World Health Organization, other government bodies and associations, etc.

* Significant (*p* < 0.05).

Almost two-thirds of the participants were educated to the university level (65.6%), with no difference between the vegetarian and non-vegetarian groups. In addition, more than half of the participants were students (54.9%). The majority of the participants studied or worked inside Saudi Arabia (83.0%). The monthly income of about one-third of the participants was less than 2000 Saudi riyals (<533 U.S. dollars; 34.6%), with a higher income for the participants who reported being non-vegetarian (*p* < 0.001). In terms of field of study, participants with a scientific background were more likely to be vegetarian (45.6% vs. 41.8%). However, participants with a nutrition background were more likely to be non-vegetarian (7.7% vs. 2.2%).

There were significant differences between the vegetarian and non-vegetarian groups in terms of BMI values, with the vegetarian participants being more likely to be underweight (16.5% vs. 7.4%), more likely to be of normal weight (66.9% vs. 48.4%), less likely to be overweight (9.6% vs. 27.0%), and less likely to be obese (7.0% vs. 17.2%). Medical diagnoses were reported by 37.2% of the participants, with a higher percentage in the non-vegetarian group than in the vegetarian group (40.6% vs. 32.4%). There were no significant differences in terms of smoking status, and participants in the vegetarian group were more likely than those in the non-vegetarian group to be moderately active (40.0% vs. 30.6%) or very active (21.6% vs. 9.6%) (*p* < 0.001). The vegetarian group also had a higher percentage of participants reporting a history of food allergies (22.3% vs. 15.8%, *p* = 0.010).

Participants in the vegetarian group were more likely than those in the non-vegetarian group to describe their diet as excellent or very good (12.5 and 26.1% vs. 4.3 and 8.3%, respectively; *p* < 0.001). The groups also differed in terms of dietary supplement use (*p* < 0.001), with the vegetarian group more likely to report using supplements, especially vitamin B12 (33.3.0% vs. 3.3%). Significant differences were also observed in terms of dietary information sources, with vegetarians more likely to rely on books and magazines (24.2% vs. 8.1%), the internet (77.2% vs. 50.8%), and social media (62.1% vs. 34.1%). Almost one-third of the participants had vegetarian relatives or friends (36.8%), and this was more common within the vegetarian group than within the non-vegetarian group (45.6% vs. 31.5%, *p* < 0.001).

### 3.2 Awareness and perceived understanding of vegetarian diets

Table 2 shows the awareness and perceived understanding of vegetarian diets. All of the study participants reported that they had heard of vegetarianism. A higher proportion of the vegetarian group reported good to excellent understanding of vegetarian diets compared with the non-vegetarian group (99.5% vs. 83.5%; *p* < 0.001).

**Table 2:** Awareness and perceived understanding of vegetarian diets (*n* = 998).

	Overall (*n* = 998)	Vegetarian *n* (%)^a^ 417 (41.8)	Non-vegetarian *n* (%)^a^ 581 (58.2)	*p* value^b^
Have you heard of vegetarian diets?				
Yes	998	417 (41.8)	581 (58.2)	–
No	0	0 (0.0)	0 (0.0)	
Do you understand what the term vegetarian diets means?				
No to moderate understanding	98 (9.8)	2 (0.5)	96 (16.5)	* < 0.001
Good to excellent understanding	900 (90.2)	415 (99.5)	485 (83.5)	

a Data are presented as numbers and percentages.

b Pearson’s chi-square tests.

### 3.3 Awareness of and adherence to dietary guidelines

Table 3 shows the participants’ self-reported awareness of and adherence to four well-established dietary guidelines. Only 28.6% of the participants were aware of Healthy Vegetarian Patterns and only 9.2% followed these guidelines, with a higher proportion of followers among the vegetarian group (71.7% vs. 28.3%; *p* < 0.001). Only 13.7% of the participants were aware of the Healthy Food Palm and only 4.4% followed it, with a higher proportion of followers among the non-vegetarian group (79.5% vs. 20.5%; *p* = 0.003). There is no significant difference in MyPyramid awareness between the two groups, with majority of the participants were aware of MyPyramid (92.8%), although only 28.0% followed it, with a higher proportion of followers was among the non-vegetarian group (67.7% vs. 32.2%; *p* < 0.001). Finally, almost half of the participants were aware of My Plate (46.7%) and 22.3% followed it, with no significant differences between the two groups.

**Table 3:** Awareness of and adherence to dietary guidelines (*n* = 998).

	Overall *n* = 998	Vegetarian *n* (%)^a^ 417 (41.8)	Non-vegetarian *n* (%)^a^ 581 (58.2)	*p* value^b^
Have you heard about healthy vegetarian patterns?				
Yes	285 (28.6)	140 (33.6)	145 (25.0)	*0.003
No	713 (71.4)	277 (66.4)	436 (75.0)	
Do you follow this guideline?				
Yes	92 (9.2)	66 (15.8)	26 (4.5)	* < 0.001
No	906 (90.8)	351 (84.2)	555 (5.5)	
Have you heard about the healthy food palm?				
Yes	137 (13.7)	46 (11.0)	91 (15.7)	*0.036
No	861 (86.3)	371 (89.0)	490 (84.3)	
Do you follow this guideline?				
Yes	44 (4.4)	9 (2.2)	35 (6.0)	*0.003
No	954 (95.6)	408 (97.8)	546 (4.0)	
Have you heard about MyPyramid?				
Yes	926 (92.8)	391 (93.8)	535 (92.1)	0.311
No	72 (7.2)	26 (6.2)	46 (7.9)	
Do you follow this guideline?				
Yes	279 (28.0)	90 (21.6)	189 (32.5)	* < 0.001
No	719 (72.0)	327 (78.4)	392 (67.5)	
Have you heard about My Plate?				
Yes	466 (46.7)	210 (50.4)	256 (44.1)	0.049
No	532 (53.3)	207 (49.6)	325 (55.9)	
Do you follow this guideline?				
Yes	223 (22.3)	102 (24.5)	121 (20.8)	0.174
No	775 (77.7)	315 (75.5)	460 (79.2)	

a Data are presented as numbers and percentages.

b Pearson’s chi-square tests.

### 3.4 Estimating the consumption frequency of various food items

As shown in Table 4, significant differences were observed between the vegetarian and non-vegetarian groups in terms of consumption of most of the examined food items, with the only exceptions being fruit juice and wholemeal/mixed-grain bread. The participants in the vegetarian group were more likely than those in the non-vegetarian group to consume fruit (*p* < 0.001), vegetables (*p* < 0.001), nuts (*p* < 0.001), seeds (*p* < 0.001), legumes (*p* < 0.001), and cooked cereals (*p* < 0.001). By contrast, the participants in the non-vegetarian group were more likely than those in the vegetarian group to consume white bread (*p* < 0.001), breakfast cereals (*p* < 0.001), red meat (*p* < 0.001), white meat (*p* < 0.001), fish/seafood (*p* < 0.001), eggs (*p* < 0.001), and dairy products (*p* < 0.001).

**Table 4:** Estimated consumption frequencies for various food items (*n* = 998).

Food item	Overall *n* = 998		Vegetarian *n* = 417 (41.8%)^a^			Non-vegetarian *n* = 581 (58.2%)^a^			*p* value^b^
	Median^c^	IQR^c^	Median^c^	IQR^c^	Mean rank	Median^c^	IQR^c^	Mean rank	
Fruit	3	3–5	4	3–5	564.8	3	2–4	452.7	<0.001
Vegetables	5	3–5	5	5–5	615.1	4	3–5	416.6	<0.001
Nuts	3	2–4	3	2–5	583.8	3	2–3	439.0	<0.001
Seeds	2	2–4	3	2–5	640.8	2	2–3	398.1	<0.001
Legumes	3	3–4	4	3–5	631.5	3	2–4	404.7	<0.001
Fruit juice	2	2–3	2	2–3	511.4	2	2–3	491.0	0.243
White bread	3	2–5	3	1–4	409.9	4	2–5	563.8	<0.001
Wholemeal/mixed-grain bread	4	3–5	4	3–5	492.6	4	3–5	504.5	0.507
Breakfast cereals	2	2–3	2	1–3	452.7	2	2–3	533.1	<0.001
Cooked cereals	4	2.7–5	4	3–5	545.5	3	2–4	466.5	<0.001
Red meat	2	1–3	1	1–1	226.5	3	2–4	695.4	<0.001
White meat	2	1–4	1	1–1	220.0	4	3–4	700.1	<0.001
Fish/seafood	2	1–2	1	1–1	258.5	2	2–3	672.5	<0.001
Eggs	2	1–2	1	1–1	248.7	4	3–4	679.5	<0.001
Dairy products	3	1–4	1	1–2	283.5	4	3–5	654.5	<0.001

a Data are presented as numbers and percentages.

b Mann–Whitney test; *p* < 0.05 was regarded as significant (*).

c Data scores represent the reported frequency of consumption in the last 6 months (daily = 5; 5–6 days per week = 4; 1–4 days per week = 3; less than once per week = 2; or never = 1) and are presented as the medians and interquartile ranges (IQRs).

### 3.5 Psychological constructs and health beliefs of the TPB model

Table 5 presents the results obtained from the TPB model, highlighting the attitude, subjective norms, and perceived behavioral control over vegetarian behavior among the study participants. Significant differences were observed between the vegetarian and non-vegetarian groups in terms of intention, attitude, subjective norms, and perceived behavioral control. The vegetarian group displayed a higher likelihood of intention to follow a vegetarian diet compared with the non-vegetarian group (*p* < 0.001). The attitude composite score and its subtypes were also significantly higher among the vegetarian group than among the non-vegetarian group (*p* < 0.001). Furthermore, the subjective norms composite score and its subscale for friends were higher among the vegetarian group than among the non-vegetarian group (*p* = 0.024 and *p* = 0.034, respectively). However, family, health experts, and colleagues displayed low median scores with no significant differences between groups, indicating a supportive environment where neither group feels considerable pressure from family expectations, the workplace, or health professionals. Finally, the perceived behavioral control composite score and its subscales were significantly higher among the vegetarian group than among the non-vegetarian group (*p* < 0.001).

**Table 5:** Theory of planned behavior (TPB) constructs and their corresponding health beliefs stratified by vegetarian status (*n* = 998).

Main TPB constructs and their related health beliefs	Range	Overall *n* = 998^a^		Vegetarian 417 (41.8%)^a^			Non-vegetarian 581 (58.2%)^a^			*p* value^b^
		Median	IQR	Median	IQR	Mean rank	Median	IQR	Mean rank	
Intention to follow a vegetarian diet^c^	1 to 2	0	0–1	1	0–1	587.8	0	0–1	436.2	<0.001
Attitude composite score^d^^,^^g^	3 to 21	5.33	4–6.67	6.67	6–7	733.2	4	3.67–5.33	331.8	<0.001
Vegetarianism rated bad to good	1 to 7	5	4–7	7	6–7	715.9	4	4–5	344.2	<0.001
Vegetarianism rated harmful to beneficial	1 to 7	6	4–7	7	6–7	709.0	4	4–6	349.2	<0.001
Vegetarianism rated unpleasant to pleasant	1 to 7	5	4–7	7	6–7	724.0	4	3–5	338.4	<0.001
Subjective norms composite score^e^^,^^g^	4 to 28	1.5	1–3	1.75	1–3	522.9	1.5	1–2.75	482.7	0.024
Friends	1 to 7	1	1–3	1	1–4	519.4	1	1–3	485.2	0.034
Family	1 to 7	1	1–3	1	1–3	496.5	1	1–3	501.6	0.749
Health experts	1 to 7	1	1–4	1	1–4	511.7	1	1–4	490.8	0.222
Colleagues	1 to 7	1	1–3	1	1–3	505.6	1	1–3	495.1	0.507
Perceived behavioral control composite score^f^^,^^g^	3 to 21	4.33	3–6.33	6.33	5.33–7	745.7	3.67	2.67–4.33	322.8	<0.001
Personal control of following a vegetarian diet	1 to 7	5	3–7	7	6–7	724.0	4	2–5	338.4	<0.001
Capability of following a vegetarian diet	1 to 7	5	3–7	7	6–7	741.3	3	2–5	325.9	<0.001
Ease of following a vegetarian diet	1 to 7	4	3–6	6	5–7	695.4	3	3–4	358.9	<0.001

a Data are presented as numbers and percentages.

b Mann–Whitney tests.

c The possible range for intention items was dichotomous, with participants answering ‘yes’ or ‘no’.

d Attitude items were scored between one and seven (from ‘bad’ to ‘good’, from ‘harmful’ to ‘beneficial’, and from ‘unpleasant’ to ‘pleasant’), with higher scores reflecting a positive attitude to following a vegetarian diet.

e Subjective norms items were scored between one and seven (from ‘not at all’ to ‘to a very great extent’), with higher scores reflecting greater social pressure to following a vegetarian diet.

f Perceived behavioral control items were scored between one and seven (from ‘very little control’ to ‘extreme/complete control’, from ‘not very capable’ to ‘very capable’, and from ‘very difficult’ to ‘very easy’), with higher scores reflecting a greater level of control over the target behavior.

g For each category, the composite score is the sum of all of the beliefs in that category.

### 3.6 Path analysis of vegetarian behavior

**Figure 1:**
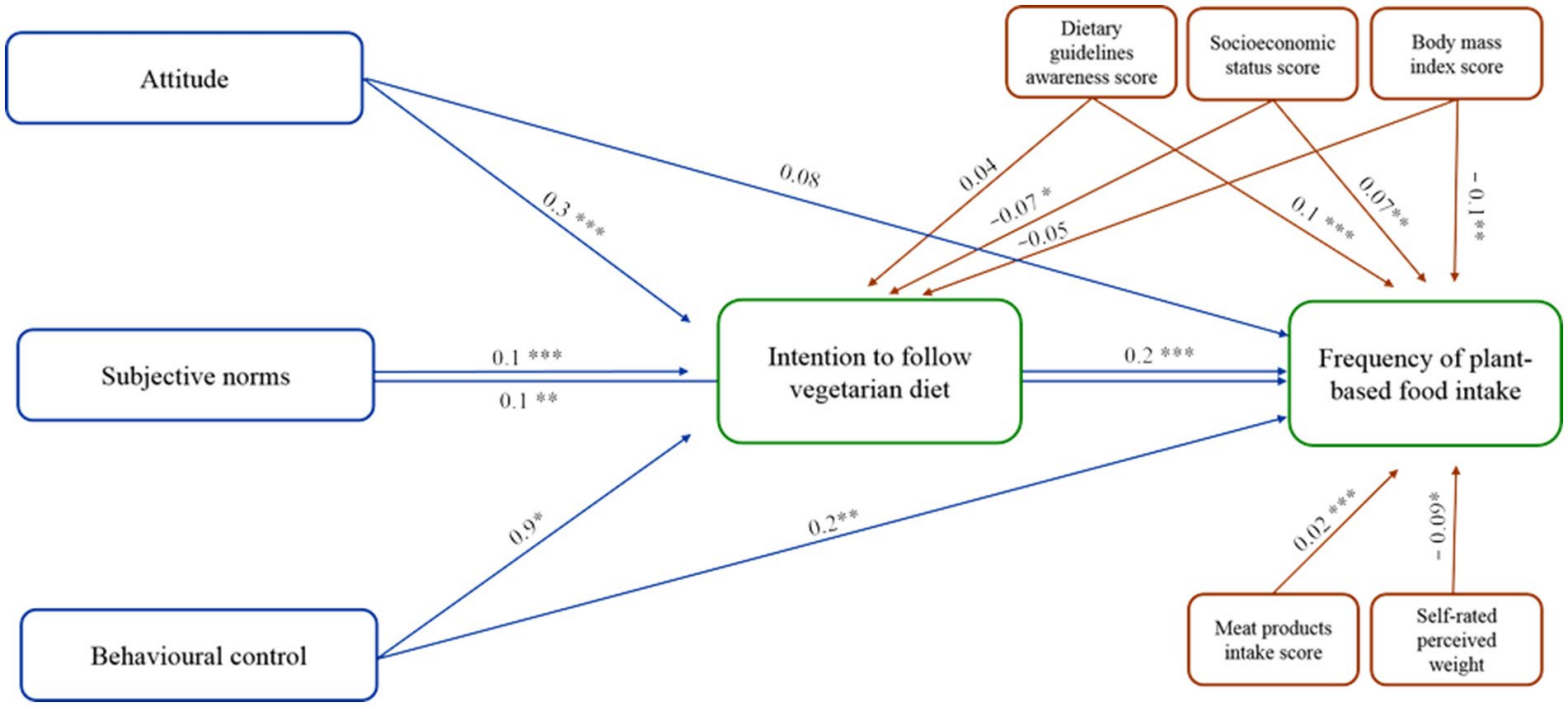
Path analysis of the TPB constructs with their corresponding health beliefs, dietary guideline awareness score, socioeconomic status index, body mass index score, self-rated perceived weight, and meat product intake score to explain the intention and behavior of more frequently consuming plant-based food items. The values are the standardized *β*-coefficients; *p < 0.05, **p < 0.01, ***p < 0.001.

The structural equation modeling path analysis method was applied to test the TPB psychological constructs, dietary guideline awareness score, socioeconomic status index, body mass index score, self-rated perceived weight, and meat product intake score to explain the intention and behavior of more frequently consuming plant-based food items. The path model fit with the implied path was assessed and the resulting goodness of fit revealed that the path model exhibited an excellent fit for the observed data (CFI = 0.982, TLI = 0.945, and NFI = 0.977; values of these goodness of fit indices exceeding 0.945 are indicative of good model fit). In addition, the RMSEA value of 0.062 (90% CI: 0.047–0.077) confirmed the overall goodness of fit of the path model with the data, while the PCLOSE value of 0.980 was congruent with a non-significant chi-square adjusted test of goodness of fit (CMIN/DF = 1.635, *p* value = 0.082). Furthermore, the path model requested some covariances be considered between the independent (exogenous) predictor variables, and these correlations were allowed and estimated (Appendix A). The decision to allow these covariances was based on their theoretical soundness alongside their intercorrelations when tested with the bivariate correlation method (Appendix B). Appendix C provides the main standardized regression coefficients estimated by the path model shown in Figure 1.

#### 3.6.1 Intention to follow a vegetarian diet

Analysis of the path model revealed that the attitude score had a significant positive impact on the intention to follow a vegetarian diet (standardized *β*-coefficient = 0.298, *p* < 0.001), which mean that females with positive attitudes toward vegetarianism are more likely to show an intention to adopt a vegetarian diet. In addition, the subjective norms score was significantly and positively correlated with the intention to follow a vegetarian diet (standardized *β*-coefficient = 0.102, *p* < 0.001), indicating that females who perceive stronger social support are more likely to intend to adopt a vegetarian diet. The perceived behavioral control composite score was also significantly and positively correlated with the intention to follow a vegetarian diet (standardized *β*-coefficient = 0.086, *p* = 0.037). This suggested that females who perceive greater control over their ability to adopt a vegetarian diet are more likely to intend to follow a vegetarian diet. However, the dietary guideline awareness score did not converge significantly on the intention to follow a vegetarian diet (standardized *β*-coefficient = −0.04, *p* = 0.141), which implies that awareness of dietary guidelines does not strongly affect females’ intentions to adopt a vegetarian diet. The path model also indicated that the socioeconomic status index had a negative correlation with the intent to follow a vegetarian diet (standardized β-coefficient = −0.073, *p* = 0.018), which mean that females of lower socioeconomic status are more likely to intend to adopt a vegetarian diet. Similarly, the body mass index score was not significantly correlated with the intention to follow a vegetarian diet (standardized *β*-coefficient = −0.05, *p* = 0.091). Hence, BMI does not have a strong influence on females’ intentions to adopt a vegetarian diet (Figure 1).

#### 3.6.2 Frequency of plant-based food intake

Analysis of the path model indicated that the intention to follow a vegetarian diet was significantly and positively correlated with the frequency of plant-based food intake score (standardized *β*-coefficient = 0.206, *p* < 0.001). This indicates that females who intended to follow a vegetarian diet perceived that they consumed significantly higher amounts of plant-based food. The attitude score was correlated positively but not significantly with the frequency of plant-based food intake score (standardized *β*-coefficient = 0.076, *p* = 0.08). This suggests that while there is a tendency for more positive attitudes to be associated with more frequent plant-based food consumption, the evidence is not strong enough to confirm a significant connection. The subjective norms score displayed a significant positive correlation with the frequency of plant-based food intake score (standardized *β*-coefficient = 0.09, *p* = 0.002), indicating that as the females’ mean perceived subjective norms score rises, their mean intake of plant-based food tends to increase accordingly. The perceived behavioral control composite score was significantly and positively correlated with the frequency of plant-based food intake score (standardized *β*-coefficient = 0.245, *p* = 0.018), showing that females who feel they have greater control over their dietary choices are likely to consume more plant-based food. In addition, the dietary guideline awareness score had a significant and positive effect on the frequency of plant-based food intake score (standardized β-coefficient = 0.146, *p* < 0.001), suggesting that as females’ awareness of dietary guideline increases, their average intake of plant-based food also tends to rise incrementally. The socioeconomic status index was significantly and positively correlated with the frequency of plant-based food intake score (standardized *β*-coefficient = 0.074, *p* = 0.016). Hence, females with a higher socioeconomic status may have perceived a significantly higher intake of plant-based food, on average. Furthermore, the path model analysis findings revealed that the body mass index score had a direct significant and negative effect on the frequency of plant-based food intake score (standardized *β*-coefficient = −0.129, *p* = 0.003). Therefore, females with a higher BMI tended to consume significantly less plant-based food. The self-rated perceived weight score was significantly and negatively correlated with the frequency of plant-based food intake score (standardized β-coefficient = −0.098, *p* = 0.021), indicating that females who perceived themselves as having higher weight tended to consume less plant-based food on average, according to the analysis model. Finally, the mean self-rated meat product intake score was correlated significantly and positively with the frequency of plant-based food intake score (standardized β-coefficient = 0.244, *p* value < 0.001). As the meat product intake score increased, the mean intake of plant-based food also tended to rise significantly (Figure 1).

## 4 Discussion

To the best of our knowledge, this is the first study to explore the factors influencing the intention to follow a vegetarian diet among Saudi females. This study is unlike any other in the Saudi community and we anticipate that our findings will contribute to a broader global understanding of people’s intentions to follow vegetarian diets. In turn, this should assist with the development of a comprehensive approach for identifying the motivations and social influences that can be utilized to design programs and interventions to promote healthier eating habits and effective behavioral changes leads to a balanced diet.

The characteristics of the vegetarian group observed in the present work are consistent with previous studies performed in Saudi Arabia and other countries, which reported that vegetarian participants were typically younger, single, students, of lower income, of normal weight, and moderately to very active compared with non-vegetarian participants (2, 9, 10, 27, 28, 41, 50, 51).

Overall, the findings of the TPB model revealed positive associations between attitude, subjective norms, and perceived behavioral control and the intention to follow a vegetarian diet. However, compared with perceived behavioral control, attitude and subjective norms showed stronger associations with vegetarianism adoption. This could be due to several reasons, including the limited acceptability of cultural vegetarian recipes, as most traditional dishes contain meat products. Consequently, individuals may feel they lack the recipes necessary to adopt a vegetarian diet. In addition, if individuals are not familiar with preparing tasty vegetarian traditional dishes that can compete with the appealing taste of meat, they may feel less confident in their ability to follow a vegetarian diet. Furthermore, individuals may feel less able to adhere to a vegetarian diet owing to religious holidays that involve traditional meat dishes, thereby undermining their perceived ability to adopt a vegetarian lifestyle.

A previous meta-analysis of the TPB model and healthy eating behavior revealed associations with psychological factors, with the strongest association with attitude, followed by subjective norms and perceived behavioral control (35). This information may therefore provide nutritional program designers with useful guidelines for developing effective dietary interventions. Gifford et al. reported a negative correlation between social norms and the intention of following a plant-based diet (37). Perhaps as a result of the influence of peer pressure, individuals who conform to social norm may find it harder to evaluate the relevant of theses norms in their decisions. However, Severijns et al. (52) reported that social norms did not influence meat consumption, while social contacts may have influenced diet through their attitude, information, and perceived behavioral control. Nonetheless, participants may not recognize these indirect effects of their peers on their dietary choices. In addition, although this study did not assess the sociocultural barriers to the adoption of vegetarian diets by women in Saudi Arabia, there is no doubt that these barriers affect individuals’ dietary behaviors and preferences. To our knowledge, no study has explored the sociocultural factors that influence the adoption of vegetarian diets among women in Saudi Arabia. However, a study by Azhar et al. (2) reported some barriers faced by women in Saudi Arabia in this context, including the prevalence of meat consumption during holidays, special occasions, and family gatherings, as well as the cultural expectation of showing generosity by providing meat on these occasions. Therefore, refusing to consume the offered meat-based dishes is considered impolite in Saudi culture, making vegetarianism difficult to adopt (2). Cost, sensory enjoyment of meat, and insufficient cooking skills have also been reported as barriers to vegetarianism for women in Saudi Arabia (2). Furthermore, external factors such as government policies and accessibility to vegetarian products could also affect women’s dietary behaviors and preferences (53). To date, there are no government policies in place to ensure that women who follow a vegetarian diet are supported in achieving optimal nutrition. Additionally, there is a lack of variety in balanced vegetarian dishes available in the market (2). Future research is necessary to address these sociocultural barriers, which will help identify the most significant factors and enable the implementation of effective health promotion strategies for individuals who decide to follow a vegetarian diet.

In the present study, there was a significant difference between the vegetarian and non-vegetarian groups in terms of attitude, subjective norms, and perceived behavioral control. The vegetarian participants rated a vegetarian diet as better, more beneficial, and more pleasant compared with the non-vegetarian participants. This may be attributable to the fact that people tend to have positive attitudes and beliefs towards their own dietary behaviors and negative attitudes and beliefs towards dietary behaviors that differ from their own (54). The positive attitudes of the participants toward vegetarianism can be observed in various real-life decisions. For example, individuals with favorable attitudes may be more willing to improve their cooking skills to try new plant-based recipes at home, seek information and resources that support vegetarianism, and join community events focused on cooking and nutrition workshops and seminars. Additionally, these positive attitudes can influence long-term dietary modifications, facilitating a transition to a vegetarian diet that they can maintain over time. The influence of friends was greater among the vegetarian participants than among the non-vegetarian participants. This may be a consequence of the majority of the study population being young, and it suggests that friendships are influential in shaping individual dietary behaviors (55, 56). Furthermore, in accordance with previous studies conducted in Saudi Arabia, the United Kingdom, Greece, and India, we found that younger adults tended to adopt a vegetarian diet more frequently than older adults (2, 27, 57). Hence, older individuals may be less likely to change certain behaviors (2, 58). On the other hand, the expectations from family, health experts, and colleagues showed no significant differences between groups, which indicates a supportive environment where neither group feels considerable pressure regarding their dietary preferences. Future research could assess how supportive environments improve individual well-being and resilience across various cultural and demographic contexts. Finally, perceived behavioral control, including personal control, capability, and ease of following a vegetarian diet, was higher among the vegetarian group. Previous studies have demonstrated that perceived behavioral control is often a significant predictor of lower meat intake and the adoption of a vegetarian diet (59, 60). Therefore, perceived behavioral control may serve as a predictor for higher self-efficacy and ability to overcome barriers. However, dietary guideline awareness did not converge significantly on the intention to follow a vegetarian diet. Among the four dietary guidelines, MyPyramid was the only guideline whose awareness was high among the two groups, with no significant differences, reflecting a general familiarity among them. This could be because MyPyramid has been a part of dietary education for a longer period, which has allowed more educational outreach via schools, community programs, and public health campaigns. Therefore, enhancing public awareness and understanding of other dietary guidelines could significantly increase their familiarity and could positively influence attitudes, subjective norms, and perceived behavioral control, ultimately promoting healthier eating behaviors. Future research is needed to provide insights into how changes in dietary awareness are associated with changes in attitudes, norms, and behaviors over time, eventually informing more effective interventions. Despite this, the awareness of and adherence to dietary guidelines exhibited significant differences between the two groups, with the vegetarian group displaying higher awareness about healthy vegetarian eating guidelines and My Plate and the non-vegetarian group having higher awareness of the Healthy Food Palm. This suggests that while awareness and guideline adherence may differ between groups, this does not alter the relationships observed between the TPB constructs and intentions. Research into the associations between awareness of and adherence to dietary guidelines and the intention to follow certain dietary guidelines is complex (61). Previous studies have reported that high awareness of guidelines does not necessarily translate to adoption or adherence in practice (62). This arises from the fact that changing the eating habits of individuals is difficult because eating habits and behavior are affected by numerous physiological, psychological, social, and environmental factors (63, 64). In addition, attitudes and preferences, social context and norms, and a lack of behavioral control (perceived or actual) can cause a gap between favorable attitudes, good intentions, and desirable behavior (35, 65). For example, even if individuals are aware of the benefits of adopting a vegetarian diet, they may find it challenging to follow due to cultural pressures or the influence of family and friends’ eating habits, which often include a variety of meat-based dishes, making vegetarianism difficult to adopt. Lastly, it could beneficial for future studies to integrate more theoretical frameworks such as the Health Belief Model and Cognitive Dissonance Theory to better understand the multifaceted factors that contribute to dietary intentions. The Health Belief Model, which focuses on individuals’ perceptions of health risks and benefits determining their behaviors, highlights that dietary awareness alone may not be sufficient if individuals do not perceive significant risks associated with their current dietary habits (66). In addition, applying Cognitive Dissonance Theory can help understanding how individuals may experience discomfort when their awareness of dietary guidelines conflicts with their current dietary behaviors, leading them to justify their behaviors rather than change their eating habits (67).

The socioeconomic status index (income, education, marital status, and working status) was negatively correlated with the participants’ intention to follow a vegetarian diet. Specifically, females with a lower socioeconomic status index were found to be less able to follow a vegetarian diet. Previous studies have also reported an association between lower socioeconomic status and a decreased intake of plant-based foods (50, 66, 67). In this study, most of the participants were younger; this group often follows the trend of vegetarian diets, especially with a focus on weight loss and health benefits. In addition, in the context of Saudi culture, the negative correlation between higher socioeconomic status and the intention to adopt vegetarianism among the study participants could be attributed to various cultural factors related to cost, accessibility, and perceptions of vegetarianism. While those with higher socioeconomic status generally have access to a variety of food options, they may struggle to find culturally acceptable vegetarian alternatives, which can hinder their intention to adopt a vegetarian diet. Additionally, individuals with higher socioeconomic status may have greater access to high-meat-rich and gourmet dishes as symbols of wealth. The perception that vegetarian diets are less luxurious or fulfilling can deter individuals from exploring vegetarian options, even when those options may be more affordable, leading them to prefer premium meat products or gourmet meals. However, studies conducted in Northern Europe and North America have reported that, compared with meat-eaters, vegetarians are more likely to belong to higher socioeconomic categories. It is possible that individuals of higher socioeconomic status are more likely to be health-conscious and therefore adopt a vegetarian diet (68).

In the present study, a significant difference was observed between the two groups, with the vegetarian group having a lower income. Previous studies have reported diverse findings, indicating a complex interplay between income and vegetarian diets. Some studies reported an association between a higher income and a vegetarian diet, whereas other studies suggested that higher-income respondents viewed vegan diets as less tasty (69). However, in the present study, the non-vegetarian group displayed a higher educational level. This is consistent with a study by Azhar et al., who reported that higher education is associated with decreased vegetarianism (2). The association between income and following a vegetarian diet appears to vary considerably depending on the country and is highly dependent on cultural habits and social background, which may explain the observed variation between countries (72). For example, a German study found no association between following a vegetarian diet and income (72), whereas French and Canadian studies reported a link between low income and following a vegetarian diet (68, 73). However, other studies have reported a positive link between a higher level of education and the adoption of a vegetarian diet (74). This could be explained by individuals with a higher educational level being healthier (75, 76) and/or more environmentally conscious.

In the present study, the marital status also differed between the two groups, with a higher proportion of the vegetarian group being single. This is consistent with the work by Azhar et al., which found a significant association between being a vegetarian and marital status, with more single people following a vegetarian diet (2).

The body mass index score was not significantly correlated with the intention to follow a vegetarian diet. However, in the present study, females with a lower BMI and a normal self-rated perceived weight tended to follow a vegetarian diet more than those in the other groups. This shows that weight perception could influence dietary choices, and how individuals view their own weight could be as important as their BMI in this context. Individuals who perceive themselves as having a healthy weight may feel more inspired to follow a vegetarian diets, which has been reported to aligned with health ideals, while individuals with higher negative self-perceptions may struggle to follow vegetarian (77, 78). Numerous studies have described the effect of a vegetarian diet on losing weight, and the prevalence of a vegetarian diet was reported to be greater among participants with a normal weight (79). This may be attributable to vegetarian diets often being lower in calories and saturated fat and higher in dietary fiber, which may support weight loss and in turn reduce obesity (80). Future studies are needed to explore the intention of obese people who follow a vegetarian diet.

This study has some limitations that should be acknowledged. First, our collection of data through an online approach using convenience sampling (snowball sampling) introduces selection bias can be considered a limitation because it does not allow for proper representation of the overall population and limits the study’s generalizability as the socio-demographic data of our study do not reflect the structure of the entire females in Saudi Arabia. However, convenience sampling was selected as the most time- and cost-effective method to gather responses from a large population (81). Additionally, the cross-sectional design of our study restricts our ability to describe actual behavioral changes after the survey. Second, our measurement of food intake was limited to frequency and did not consider the consumption amount. Third, the vegetarian or non-vegetarian status of the participants was self-reported; thus, both recall and social acceptability biases are possible. Fourthly, the aims of our study were explorational rather than directed at determining prevalence. Therefore, further studies should be conducted to examine the prevalence of vegetarianism in Saudi Arabia. Lastly, this study did not investigate the reasons females choose to adopt vegetarian diets, which may limit the understanding of the factors influencing their dietary decisions and motivations.

## 5 Conclusion

The current study provides a better understanding of vegetarianism among Saudi females based on the TPB model. The results demonstrate significant associations between attitude, subjective norms, and perceived behavioral control influences and the behavioral intention to adopt a vegetarian diet. It is important to understand these predictors, as they could assist with formulation of public health policies, such as the development of national dietary guidelines that incorporate recommendations for balanced vegetarian diets. In addition, collaboration with the food industry to create fortified and balanced vegetarian products and meals will support individuals in making healthier dietary choices and improving overall public health outcomes. Furthermore, the study findings could encourage the design of effective nutritional education strategies to support the vegetarian population in follow a balanced vegetarian diet. Such strategies may include workshops and cooking classes to teach meal preparation, online resources that provide comprehensive information, and community outreach programs to engage diverse populations. This could help with the prevention and management of certain chronic diseases, while limiting the potential health risk factors associated with following a vegetarian diet. Further studies are warranted to explore the personal motivations of individuals adopting a vegetarian diet such as reasons related to health or environmental/ economic concerns. Considering of the psychological and social factors influencing dietary choices will be essential for developing targeted interventions. In addition, longitudinal studies to assess the long-term health impact of a vegetarian diet, taking into account different sociodemographic groups, are important to enhance the understanding of the effectiveness and safety of vegetarian diets as well as ensure that dietary guidelines are evidence-based and relevant to diverse communities. Lastly, conducting a nationwide survey to estimate the prevalence and intention to follow vegetarianism using a stratified sampling method (probability sampling) across diverse populations, while considering national census data, is essential. The data from this survey not only provide a clearer picture of dietary preferences but also help inform public health policies and nutritional education strategies.

## Data Availability

The original contributions presented in the study are included in the article, further inquiries can be directed to the corresponding authors.
