# Supplementary material for: Using the theory of planned behaviour to understand the adoption of vegetarianism among females in Saudi Arabia
Source: Front Public Health. 2025 May 14;13:1566712. doi: 10.3389/fpubh.2025.1566712 (PMC12116532; doi:10.3389/fpubh.2025.1566712)
Supplement: Supplementary file 1 [file Table_1.DOCX]

Supplementary Material

Using the Theory of Planned Behaviour to Understand the Adoption of Vegetarianism among Females in Saudi Arabia

Appendix A

**Table A1.** Path model standardized bivariate correlations for following a vegetarian diet.

| **Variable A** | **Variable B** | **Standardized correlation coefficient** | ***p* value** |
| --- | --- | --- | --- |
| Attitude composite score | Dietary guideline awareness score | 0.049 | 0.025 |
| Body mass index score | Self-rated perceived weight | 0.743 | <0.001 |
| Attitude composite score | Subjective norms composite score | 0.147 | <0.001 |
| Body mass index score | Meat product intake score | 0.101 | <0.001 |
| Attitude composite score | Meat product intake score | −0.615 | <0.001 |
| Self-rated perceived weight | Meat product intake score | 0.052 | <0.001 |
| Dietary guideline awareness score | Meat product intake score | −0.051 | <0.001 |
| Meat product intake score | Socioeconomic status index | 0.284 | <0.001 |
| Self-rated perceived weight | Socioeconomic status index | 0.172 | 0.025 |
| Attitude composite score | Socioeconomic status index | −0.215 | 0.034 |
| Body mass index score | Socioeconomic status index | 0.244 | <0.001 |
| Subjective norms composite score | Socioeconomic status index | 0.065 | 0.028 |
| Attitude composite score | Perceived behavioural control composite score | 0.720 | <0.001 |
| Meat product intake score | Perceived behavioural control composite score | −0.625 | <0.001 |
| Socioeconomic status index | Perceived behavioural control composite score | −0.192 | <0.001 |
| Subjective norms composite score | Perceived behavioural control composite score | 0.130 | <0.001 |

Appendix B

**Table A2.** Bivariate Pearson’s correlations between the measured variables.

|  | Frequency of plant-based food intake score | Dietary guideline awareness score | Meat product intake score | Attitude composite score | Subjective norms composite score | Perceived behavioural control composite score | | Age | Body mass index score | Self-rated perceived weight | Socioeconomic status index |
| --- | --- | --- | --- | --- | --- | --- | --- | --- | --- | --- | --- |
| Frequency of plant-based food intake score | 1.000 |  |  |  |  |  |  | |  |  |  |
| Dietary guideline awareness score | 0.186^**^ |  |  |  |  |  |  | |  |  |  |
| Meat product intake score | −0.057 | −0.255^**^ |  |  |  |  |  | |  |  |  |
| Attitude composite score | 0.223^**^ | 0.220^**^ | −0.628^**^ |  |  |  |  | |  |  |  |
| Subjective norms composite score | 0.162^**^ | −0.028 | −0.040 | 0.169^**^ |  |  |  | |  |  |  |
| Perceived behavioural control composite score | 0.265^**^ | 0.219^**^ | −0.644^**^ | 0.724^**^ | 0.154^**^ |  |  | |  |  |  |
| Age | −0.040 | −0.192^**^ | 0.358^**^ | −0.253^**^ | 0.064^*^ | −0.286^**^ |  | |  |  |  |
| Body mass index | −0.213^**^ | 0.002 | 0.233^**^ | −0.149^**^ | −0.009 | −0.217^**^ | 0.371^**^ | |  |  |  |
| Self-rated perceived weight | −0.208^**^ | −0.063^*^ | 0.148^**^ | −0.096^**^ | 0.016 | −0.160^**^ | 0.248^**^ | | 0.348^**^ |  |  |
| Socioeconomic status index | −0.013 | −0.063^*^ | 0.310^**^ | −0.247^**^ | 0.051 | −0.241^**^ | 0.779^**^ | | 0.284** | 0.202^**^ |  |

* Significant correlation at the 0.05 level (two-tailed). ** Significant correlation at the 0.01 level (two-tailed).

Appendix C

**Table A3.** Path model regression standardized coefficients.

| **Independent (predictor) variable** | **Dependent (outcome) variable** | **Standardized β-coefficient** | ***p* value** |
| --- | --- | --- | --- |
| Attitude composite score | Intention to follow a vegetarian diet | 0.298 | <0.001 |
| Subjective norms composite score | Intention to follow a vegetarian diet | 0.102 | <0.001 |
| Perceived behavioural control composite score | Intention to follow a vegetarian diet | 0.086 | 0.037 |
| Dietary guideline awareness score | Intention to follow a vegetarian diet | 0.042 | 0.141 |
| Socioeconomic status index | Intention to follow a vegetarian diet | −0.073 | 0.018 |
| Body mass index score | Intention to follow a vegetarian diet | −0.05 | 0.091 |
| Intention to follow a vegetarian diet | Frequency of plant-based food intake score | 0.206 | <0.001 |
| Attitude composite score | Frequency of plant-based food intake score | 0.076 | 0.086 |
| Subjective norms composite score | Frequency of plant-based food intake score | 0.09 | 0.002 |
| Perceived behavioural control composite score | Frequency of plant-based food intake score | 0.245 | 0.018 |
| Dietary guideline awareness score | Frequency of plant-based food intake score | 0.146 | <0.001 |
| Socioeconomic status index | Frequency of plant-based food intake score | 0.074 | 0.016 |
| Body mass index score | Frequency of plant-based food intake score | −0.129 | 0.003 |
| Self-rated perceived weight | Frequency of plant-based food intake score | −0.098 | 0.021 |
| Meat product intake score | Frequency of plant-based food intake score | 0.244 | <0.001 |

Path model overall fit: (χ^2^) = 67.11, df = 17, CMIN/DF = 4.794, *p* < 0.001. CFI = 0.982, TLI = 0.945, NFI = 0.977, RMSEA = 0.062, RMSEA 90% CI (0.047–0.077), PCLOSE=0.088.
